# The Role of Immunotherapy to Overcome Resistance in Viral-Associated Head and Neck Cancer

**DOI:** 10.3389/fonc.2021.649963

**Published:** 2021-07-16

**Authors:** Rebecca R. Pharaon, Yan Xing, Mark Agulnik, Victoria M. Villaflor

**Affiliations:** Department of Medical Oncology and Therapeutics Research, City of Hope, Duarte, CA, United States

**Keywords:** immunotherapy, head and neck cancer, oropharyngeal squamous cell carcinoma, nasopharyngeal carcinoma, viral-associated cancers, human papillomavirus, Epstein–Barr virus

## Abstract

A subset of head and neck cancers arising in the oropharynx and the nasopharynx are associated with human papillomavirus or Epstein–Barr virus. Unfortunately, limited treatment options exist once patients develop recurrent or metastatic disease in these cancers. Interest has risen in utilizing novel strategies including combination immune checkpoint inhibitors, vaccines, and adoptive cellular therapy, to improve treatment response and outcomes. Several ongoing studies are investigating the potential to overcome resistance to standard of care chemoradiation therapy with monotherapy or combination immunotherapy strategies in these viral-associated head and neck cancers.

## Introduction

Head and neck cancers (HNC) are a heterogenous group of malignancies. Historically, the risk factors for developing HNC were tobacco, betel nut, and alcohol consumption. With emerging data, chronic viral infections such has human papillomavirus (HPV) and Epstein–Barr virus (EBV) have also been associated with the development of cancer. Generally, HPV-negative HNCs are found in older individuals with a history of tobacco and alcohol use. On the other hand, HPV-positive HNCs, which develop in the oropharynx, are seen in much younger patients with associated risk factors such as sexual behavior and marijuana use. EBV-associated HNCs, which develop in the nasopharynx, are also found in younger patients often in endemic areas and are associated with risk factors such as high consumption of salt-cured foods and tobacco use. We will focus on HPV and EBV in the development of oropharyngeal squamous cell carcinoma (OPSCC) and nasopharyngeal cancer (NPC), respectively.

HPV has been implicated in cervical, oropharynx, anal, and penile cancers (1). HPV is transmitted through skin-to-skin or skin-to-mucosa contact, typically through sexual transmission. The causative link with HPV and OPC was first described in 2000 (2). The number of reported cases has risen over the past 20 years; it is presumed up to 70–80% of all OPSCC in North America and Europe are now HPV-related (3, 4). Although there are approximately 200 different HPV strains, HPV-16 makes up more than 90% of HPV-induced OPSCC (5, 6). IHC p16 staining is a surrogate marker for HPV however, ISH (*In-situ* hybridization) or PCR (polymerase chain reaction) is the gold standard for testing and can be used for confirmatory testing. Patients diagnosed with HPV-related OPSCC are considerably younger with a biphasic distribution which peaks at 30 to 55 years of age (4–6). Up to 10–25% of these patients will recur or develop metastatic disease following definitive treatment depending on tumor biology and clinical risk factors (7).

EBV has been implicated in multiple malignancies including NPC, gastric carcinoma, and lymphoma. EBV is transmitted through bodily fluids, especially saliva, as well as sexual transmission. In developed countries, NPC has been associated with smoking history; however, it is endemic in areas of China and Africa (8). EBV-encoded RNA (EBER) ISH determines if NPC is of EBV etiology. In high incidence areas, risks may be multifactorial including EBV, tobacco, diets high in preservatives and genetic predisposition (9). Patients in high risk populations to develop EBV-related NPC are generally diagnosed at 50–59 years of age (10). Approximately, 10–45% of treated NPC patients will recur. Since the landmark trial of chemoradiation followed by consolidative chemotherapy (11), there have been few new options for both curative intent and palliative treatments of NPC.

Most people who become infected with a virus clear the infection and develop no sequelae. Few become chronically infected with high risk HPV strains or EBV and those individuals may subsequently develop cancer. Immune dysfunction is implicated in development and progression of all head and neck malignancies and, given the bodies expected immune response to viral infection, this may be especially true for viral-mediated OPSCC (12). Immune therapy has emerged as a treatment to overcome dysfunction in the definitive and palliative settings for HPV- and EBV-mediated OPSCC and NPC, respectively.

## The Immune System

There are two types of immunity, innate and adaptive. The innate system is primitive, nonspecific and responds rapidly utilizing barriers that already exist in the body, examples of these are skin or cough. Cellular components of the innate system include phagocytic cells (e.g., macrophages, dendritic cells, and neutrophils) and Natural Killer (NK) cells which induce apoptosis. The adaptive immune system is acquired and involves the use of self-proteins to recognize foreign materials. Additionally, it has ability to develop memory through use of B and T lymphocytes for humoral and cell-mediated immunity. It is the adaptive system of immunity that is predominately targeted to develop immune therapies. This has been a successful approach that is furthest along in development for patients with leukemia/lymphoma, melanoma and lung cancers who have progressed on other therapies.

The tumor microenvironment (TME) consists of various components including tumor cells, endothelial cells, and immune cells such as lymphocytes, macrophages, and cytokines. In the TME, tumor cells gain control of signaling pathways using components of the TME to evade detection by the immune environment as well as promote tumor growth and metastasis. Immune dysfunction plays a role in development and progression of HNC. Specifically, T cytotoxic cells are responsible for cancer immune surveillance. Disruption of T cell response to tumor by immunosuppression in the TME or by cancer evasion mechanisms may play a role in progression of cancer (12). In viral-associated HNCs, the immune system is responsible for detecting the virus, but these viruses employ immune evasion strategies to escape detection and allow tumorigenesis. This makes the immune system a perfect target to exploit for treatment.

Potential targets of the immune system include, cytokine therapies, antibody-dependent cellular cytotoxicity (ADCC), checkpoint inhibition, vaccination, and cellular adoptive therapies. In HNC, the use of cetuximab has shown activity, which inhibits EGFR signaling and ADCC is believed to be a critical component of its response (13–15). Checkpoint inhibitor immunotherapy is an accepted paradigm for treatment in lung cancer, melanoma, and microsatellite instability (MSI)-high colorectal cancer (16–18). More recently, checkpoint inhibitors have shown promise in HNC with or without chemotherapy for treatment of metastatic disease (19). To leverage more durable response to immunotherapy in solid tumor malignancies, ongoing trials are investigating new immune checkpoint inhibitors, immunotherapy combination regimens including addition of cytokines, other checkpoint inhibitors, oncolytic virus, vaccines (VERSATILE-002 announced at the ESMO 2020 Annual Meeting), or cellular therapies such as chimeric antigen receptor therapy (CAR T) to program death ligand-1 (PD-L1) blockade.

## OPSCC, HPV-Induced

**Figure 1:**
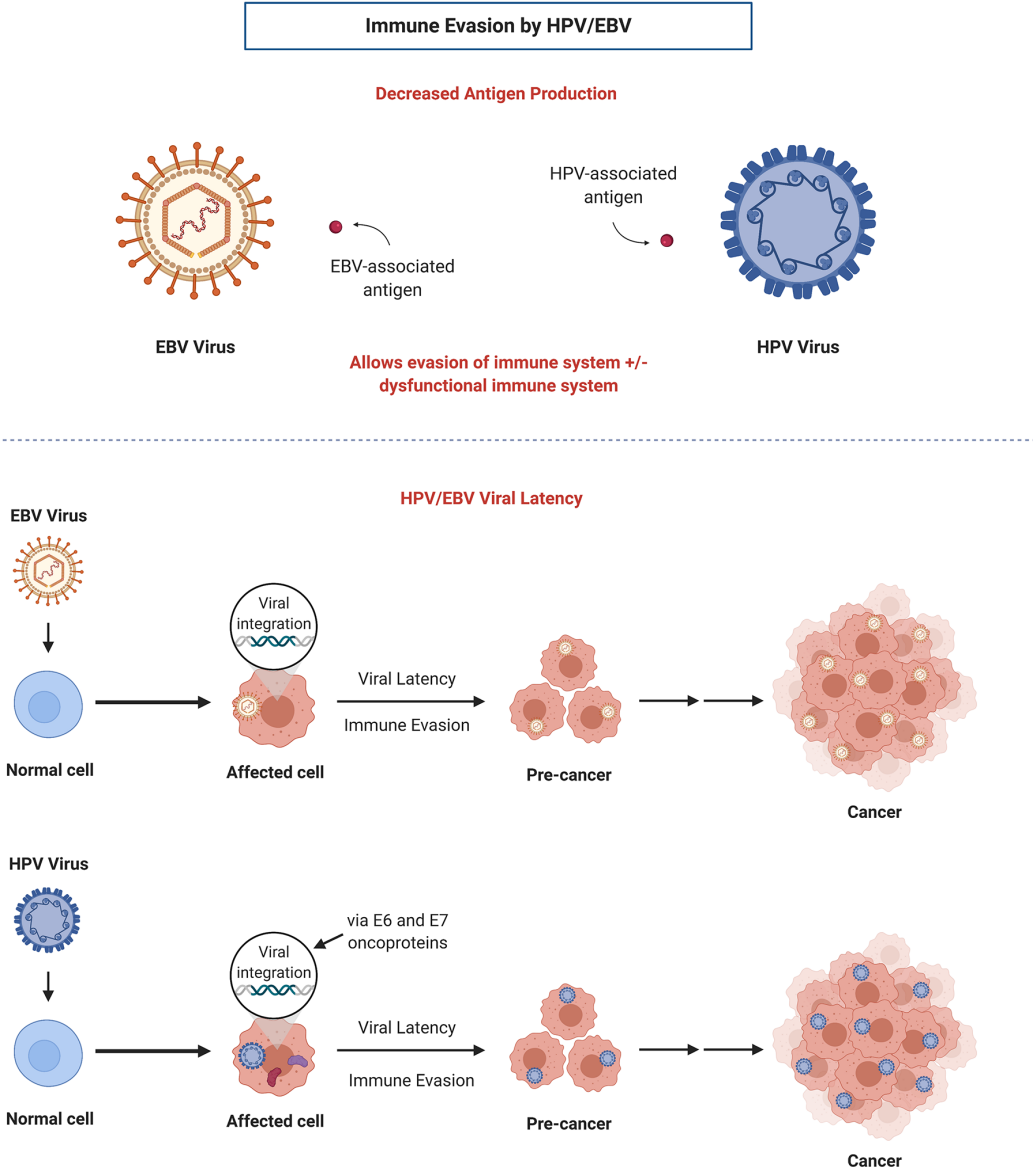
Mechanisms of immune evasion by HPV and EBV: decreased antigen production and establishment of viral latency. Adapted from “Viral Carcinogenesis”, by BioRender.com (2021). Retrieved from https://app.biorender.com/biorender-templates.

OPSCC is a subset of HNC originating in the base of tongue or tonsils which can be caused by chronic HPV infection. HPV, a prevalent viral infection, is a DNA oncovirus with numerous subtypes that have been implicated in tumorigenesis of several primary sites including the oropharynx and cervix (HPV 16 and 18). The FDA approved a HPV vaccine series targeted as a preventative measure against HPV-associated cancers (20). Majority of people exposed to HPV will not develop cancer; however, in some cases, viral HPV DNA oncogenes for E6 and E7 will integrate into the DNA of the host cell and ultimately lead to degradation and loss of p53 and RB tumor suppressor genes (21). This dysregulation of p53 and RB leads to cancer cell immortalization and uncontrolled cell proliferation. During its life cycle, HPV minimizes antigen production in order to evade recognition by the host immune system (**Figure 1**) (22). As well, oncoproteins E6 and E7 bind to immune regulator proteins to reduce and block immune responses, thus achieving immune evasion for the virus as well as the tumor, which may create a challenge when incorporating immunotherapy in treating HPV-positive OPSCC.

The incidence of HPV-associated OPSCC has risen over the past several decades while HPV-negative OPSCC has steadily declined (6). It has been previously reported that HPV-positive OPSCC is associated with better survival and overall prognosis compared to its HPV-negative counterpart due to their different etiologies (23–26). However, clinicians have found no difference in rates of development of distant metastases between HPV and non-HPV OPSCC (27). Treatment of early stage or locally advanced OPSCC generally incorporates a multi-modality approach while recurrent or metastatic OPSCC is treated with systemic therapy.

### Treatment for Locally Advanced HNC

Patients with locally advanced HNC, independent of HPV status, have been treated with multimodality treatments, often including some combination of surgery, radiation, and/or concurrent chemoradiotherapy. In HPV-mediated disease, there has been a paradigm shift to reduce long-term toxicity by de-escalating treatment recommendations. To date, the significance of immunotherapy has been realized in patients with metastatic HPV-mediated OPSCC and interest has risen in exploring its use in de-escalation strategies for definitive treatment. To date, there is little data in this indication with immune therapy. Recently, the Radiation Therapy Oncology Group (RTOG 1016) explored HPV-positive OPSCC patient outcomes and concluded that concurrent chemoradiation therapy with systemic treatment of cisplatin demonstrated superior 5-year overall survival (OS) and progression-free survival (PFS) compared to cetuximab (28). The phase III De-ESCALaTE trial confirmed these findings (29).

Most studies incorporating immune therapy with HNC have included all primary sites, including OPSCC irrespective of HPV status. Additionally, the use of response rates as primary endpoint has generated a lot of thought-provoking data. Some of these data are encouraging as response rates are high; however, OS and distant failure rates are also of concern. While patients with HPV-induced OPSCC conventionally have better survival, approximately 10–25% of patients will develop disease recurrence (30). HPV-positive patients also develop distant metastasis approximately 2–3 years later than non-HPV OPSCC patients (31). Given there may be an immune defect in these patients, it is unclear if immune therapies in the definitive indication will be sufficient, but there are ongoing trials.

#### Clinical Trials of Checkpoint Inhibitors in Locally Advanced HNC

##### Locally Advanced HNSCC, Unselected Population

Historically, patients with early or locally advanced disease have been treated with curative intent. Curative intent treatments may include surgery, radiation, and combined chemoradiation. Several clinical trials are currently underway in locally advanced HNSCC (**Table 1**). In the neoadjuvant/adjuvant setting, there are preliminary data available for two trials. Preliminary results from an ongoing phase II trial (NCT02296684) of neoadjuvant and adjuvant treatment with pembrolizumab in surgically resectable HNSCC patients showed safety, tolerability, and response to treatment although further investigation is necessary (32). Preliminary results for the IMCISION trial for 32 advanced HNSCC patients treated with neoadjuvant nivolumab monotherapy or in combination with ipilimumab (33) demonstrated 31% (9/29) near complete pathological response (≥90% pathological response) and 31% of patients 20–89% pathological response in the primary tumor specimen at resection (33). A trial combined definitive concurrent chemoradiation therapy with pembrolizumab (six doses every 3 weeks) in locally advanced HNSCC patients with high risk features of high T stage and/or nodal disease (NCT02641093). Roughly 47% (9/19) patients demonstrated a pathological response which was correlated with increased immune cell infiltration into the tumor (34).

**Table 1:** Ongoing clinical trials investigating novel immunotherapy drugs or combinations in locally advanced HNC and HPV-positive OPSCC.

Study Title	NCT	Primary Endpoint	N	Phase	Experimental	Control	Definitive treatment
IMMUNEBOOST: Feasibility and Tolerance of Nivolumab Neoadjuvant Immunotherapy in High Risk HPV Driven OPSCC	03838263	Feasibility	61	2, Randomized Multicenter	Neoadjuvant nivolumab	No neoadjuvant treatment	CRT with high dose cisplatin
Durvalumab Before Surgery in Treating Patients with Oral Cavity or OPSCC	02827838	Effect of durvalumab on local and systemic immune activation	20	2 Single center Pilot trial	Neoadjuvant durvalumab	N/A	Surgical Resection
Stereotactic Body Radiation Therapy and Durvalumab With or Without Tremelimumab Before Surgery in Treating Participants with HPV Positive OPSCC	03618134	Safety, PFS, incidence of severe adverse events	82	1b/2 Single center	Neoadjuvant stereotactic body radiotherapy and durvalumab, plus/minus tremelimumab	N/A	Surgical resection
Radiotherapy, Carboplatin/Paclitaxel and Nivolumab for High Risk HPV-related HNC	03829722	PFS	40	2 Single center	CRT plus nivolumab followed by adjuvant nivolumab	CRT with carboplatin and paclitaxel	CRT with carboplatin and paclitaxel
Testing Immunotherapy Versus Observation in Patients with HPV OPSCC	03811015	PFS and OS	744	2/3 Randomized Multicenter	Adjuvant nivolumab after chemoradiation therapy with weekly cisplatin	CRT with weekly cisplatin	CRT with weekly cisplatin
Ipilimumab, Nivolumab, and Radiation Therapy in Treating Patients with HPV Positive Advanced OPSCC	03799445	Dose-limiting toxicity, complete response rate, and PFS	180	2 Single center	Concurrent radiation therapy, nivolumab, and ipilumumab	N/A	Radiation therapy
Radiation Therapy with Durvalumab or Cetuximab in Treating Patients with Locoregionally Advanced HNC Who Cannot Take Cisplatin	03258554	Dose-limiting toxicity, PFS, and OS	523	2/3 Randomized Multicenter	Concurrent radiation therapy with durvalumab	CRT with cetuximab	CRT with cetuximab
De-intensified Radiation Therapy with Chemotherapy (Cisplatin) or Immunotherapy (Nivolumab) in Treating Patients with Early-Stage, HPV-Positive, Non-Smoking Associated OPSCC	03952585	PFS and quality of life	711	2/3 Randomized Multicenter	Concurrent reduced radiation therapy with nivolumab q 2 weeks	Concurrent reduced chemoradiation therapy with cisplatin x 2 doses	CRT
Adjuvant Therapy for High-Risk HPV 16-Positive OPSCC Patients with Durvalumab and MEDI0457 (INO-3112)	04001413	Clearance of HPV biomarkers post-treatment	66	2 Randomized Multicenter	Adjuvant durvalumab plus or minus DNA vaccine, MEDI0457	Observation	N/A
E7 TCR Cell Induction Immunotherapy for Stage II and Stage III HPV-Associated OPSCC	04015336	Feasibility	180	2 Single center	E7 TCR therapy	N/A	N/A

N/A, not applicable.

##### Locally Advanced HNSCC With HPV Subset Analysis

A phase Ib trial of pembrolizumab in combination with concurrent cisplatin-based chemoradiation therapy (NCT02586207) enrolled 59 locally advanced HNSCC patients (35). In the HPV-positive cohort (N = 34), 85.3% of patients achieved a complete response. The study demonstrated safety and tolerability of standard multi-modality treatment involving pembrolizumab as the results are similar to the expected level of response with conventional platinum-based chemoradiotherapy.

##### Locally Advanced HPV-Positive OPSCC

In viral-associated cancers such as OPSCC, CheckMate 358 examined the efficacy of nivolumab in the neoadjuvant setting and presented promising results at ESMO (NCT02488759). In the cohort of OPSCC patients enrolled in the trial, tumor reduction was seen in approximately 48% of evaluable patients (11/23, (5/10 HPV+, 6/13 HPV−)) prior to surgery (36). Final results from the CIAO (Checkpoint Inhibitors Assessment in Oropharynx Carcinoma) trial was recently published (37). Locally advanced OPSCC patients (N = 28), 24 (86%) were HPV+, were randomized 1:1 to receive durvalumab alone or combination with anti-CTLA-4 monoclonal antibody tremelimumab prior to surgery. The primary objective was to determine the impact of immunotherapy pre-treatment on CD8+ tumor infiltrating lymphocyte (TIL) count in tumor specimen. The overall response rate (ORR) was 43% in both treatment groups and 29% of patients showed a major pathologic response after treatment. The study concluded that although combination durvalumab and tremelimumab did not increase CD8+ TIL cells compared to monotherapy durvalumab, there is clinical rationale to continue investigating immunotherapy in the neoadjuvant setting. There are several recruiting clinical trials examining more combination immunotherapy strategies in early stage or locally advanced HNC and HPV-driven OPSCC in the neoadjuvant, concurrent, and adjuvant setting (38, 39) (Refer to **Table 1**).

### Treatment for Recurrent and Metastatic HNC

The first FDA approved immune therapy in HNC was single agent cetuximab. Cetuximab as a single agent has a 12.6% objective response rate and median survival of 5.9 months in patients who failed platinum-based therapy (13). With the addition of cetuximab to platinum-based therapy (EXTREME Trial), the median OS improved from 7.4 to 10.1 months (14). The EXTREME trial did establish a new standard of care; however, the survival remained poor (12).

#### Checkpoint Inhibitors in Metastatic HNC

KEYNOTE-012 trial demonstrated the efficacy of immune checkpoint inhibitors in HNC (19). The ORR was 21% and median OS was 13 months in HNC patients who had failed prior platinum therapy and who had PD-L1 combined positivity score (CPS) of 1% or greater. These findings were confirmed in the expansion cohort using fixed 3-week dosing (40). The degree of PD-L1 expression was noted to be strongly predictive of overall response, PFS and OS. The ORR was 22% and 4% for PD-L1 positive and negative patients, respectively. Additionally, HPV-positive HNC had a higher ORR of 32% compared to HPV-negative patients at 14% when treated with pembrolizumab.

Checkmate-141 trial was a similar checkpoint inhibitor trial which was ongoing at the same time as KEYNOTE-012 (41). Checkmate-141 also evaluated recurrent/metastatic HNC patients who had failed prior platinum therapy. These trials demonstrated similar findings although, cross-trial comparisons need to be interpreted with caution. The Checkmate-141 trial compared nivolumab to standard second line therapies and demonstrated that nivolumab nearly doubled 1-year OS from 16.6% with standard therapies vs 36% with nivolumab; ORR and OS were 16.6%, 5.1 months versus 36%, 7.5 months for standard therapy versus nivolumab, respectively. Additionally, exploratory analysis from the trial suggested that HPV-associated disease appeared to benefit most with response rates of 8% in HPV-negative and 15.9% in HPV-positive patients. This is interesting with both KEYNOTE and Checkmate studies suggesting HPV-positive patients have greater improvement with checkpoint inhibition then non-HPV-mediated HNC. Although this is provocative data, more work needs to be done using HPV ISH or PCR as a marker opposed to p16 used in these studies given recent data (42).

KEYNOTE-048, a phase III trial, demonstrated efficacy of pembrolizumab monotherapy or combination treatment in patients with recurrent or metastatic HNC (43). This trial revolutionized first line treatment of recurrent or metastatic HNC which was previously limited to chemotherapy and cetuximab. In the trial, patients (N = 882) were randomized to receive pembrolizumab monotherapy (N = 301), pembrolizumab and chemotherapy combination (N = 281), or cetuximab and chemotherapy combination treatment (N = 300). PD-L1 expression *via* CPS was tested and patients were stratified into groups based on CPS. The primary endpoint of median OS demonstrated significant superiority in the pembrolizumab monotherapy group compared to cetuximab and chemotherapy group in patients who exhibited a CPS of 20% or more (14.9 months vs 10.7 months, p = 0.0007) and patients who exhibited a CPS of 1% or more (12.3 months vs 10.3 months, p = 0.0086). In the overall trial population, pembrolizumab and chemotherapy combination treatment demonstrated significant improved OS compared to cetuximab and chemotherapy (13.0 months vs 10.7 months, p = 0.0034). In the subgroup of patients with CPS of 20% or more, CPS of 1% or more, and in the total patient population treated with pembrolizumab and chemotherapy, the ORR was 43%, 36%, and 36% respectively. Although pembrolizumab alone or in combination with chemotherapy did not show improved PFS in any subgroup analysis, the OS results were significant enough to transform first line standard-of-care practice in recurrent or metastatic HNSCC.

#### Ongoing Trials and Development in HNC

##### Checkpoint Inhibitors

Immunotherapy is being exploited in clinical studies of HNC and OPSCC in the recurrent or metastatic setting. Currently in recurrent and/or metastatic HNC, there are approved antibody treatments targeting PD-1 (pembrolizumab and nivolumab) (44). However, since only a small cohort of patients respond to immune checkpoint inhibitors, other strategies are under investigation to increase efficacy and response to immunotherapy.

Clinical trials on novel combination regimens with other immune checkpoint inhibitors or chemotherapy agents are currently under examination in HNC and OPSCC. Recently, the phase III EAGLE study failed to demonstrate superior OS with durvalumab monotherapy or in combination with tremelimumab compared to standard-of-care in recurrent or metastatic HNSCC patients (45). Results from a phase II trial of combination pembrolizumab and a histone deacytelase (HDAC) inhibitor, vorinostat, were recently published (46). In the recurrent or metastatic HNC arm, 25 patients were reenrolled to receive both drugs, of which the majority achieved either a partial response (32%) or stable disease (20%). The median OS was 12.6 months and the median PFS was 4.5 months, ultimately suggesting clinical activity in HNC patients however, further study is needed.

In patients with HPV-positive tumors, M7824, a bifunctional fusion protein that targets both PD-L1 and transforming growth factor-β (TGF-β) is currently under investigation. TGF-β has been previously reported to be upregulated in HPV-associated cancers (47), and thus dual targeting of PD-L1 and TGF-β should ideally produce a more durable response. Results from a phase I clinical trial investigating M7824 found an ORR of 37.5% in HPV-associated cancers (48) and it is under continued evaluation in a phase II trial (NCT03427411).

##### Vaccines

Over the past several years, scientists began to study the feasibility of combining immune checkpoint inhibitors with vaccines as a way to augment therapeutic responses. The MASTERKEY-232 phase Ib study enrolled recurrent or metastatic HNSCC patients (N = 36) to undergo treatment with pembrolizumab and talimogene laherparepvec (T-VEC), a genetically modified oncolytic viral therapy originally manufactured to treat melanoma (49). The objectives were to understand any dose-limiting toxicities, examine the safety of the combination, as well as OS and PFS. Confirmed partial responses were observed in 13.9% of patients (N = 5), and the median OS and PFS was 5.8 months and 3.0 months, respectively. The trial failed to demonstrate superior efficacy in this novel combination compared to monotherapy pembrolizumab, thus follow-up studies were discontinued. Another clinical trial combined nivolumab with an HPV-16 vaccine (ISA101) to increase therapeutic response in HPV-positive solid tumor malignancies (50). With 24 patients enrolled in the trial, the ORR was 33% and the median duration of response was 10.3 months. Median OS was 17.5 months and the median PFS was 2.7 months. Overall, this study demonstrated clinical benefit in the addition of a vaccine to immunotherapy although further studies are warranted. Currently, there is an ongoing phase II trial of anti-PD-1 monoclonal antibody cemiplimab alone or in combination with cancer vaccine ISA101b targeted at oncogenic E6 and E7 antigens from HPV-16 (NCT03669718). Another ongoing phase I/II trial is utilizing modified viruses, HB-201 and HB-202, as single vector/two-vector therapies engineered to recognize antigens specific to HPV-16 (NCT04180215).

Le Tourneau et al. presented interim results of their phase Ib/II trial investigating TG4001, a HPV vaccine targeting E6 and E7, in combination with avelumab, an anti-PD-L1 monoclonal antibody, in HPV-16 positive recurrent or metastatic solid tumors (NCT03260023) (51). The results showed increased CD8+ T cell infiltration as well as detectable vaccine responses against E6 and E7. Aggarwal et al. reported the results of a phase Ib/II study in HPV-associated recurrent or metastatic HNSCC treated with HPV DNA vaccine MEDI0457 and durvalumab (NCT03162224) (52). The trial reported an ORR of 22.2% with three each confirmed complete and partial responses. The authors also noted increased levels of peripheral HPV-specific T cells and CD8+ T cells in their treated patients. Another phase I/II trial is investigating M7824, the anti-PD-L1/TGF-β fusion protein, in combination with HPV-16 cancer vaccine PDS0101 and immunocytokine NHS-IL12 in metastatic/refractory HPV-associated solid tumor malignancies (NCT04287868). M7824 is also under examination in another phase I/II trial in combination with PRGN-2009, a novel gorilla adenovirus GAd HPV vaccine with agonist epitopes of E6 and E7 (NCT04432597), based on previously reported data that demonstrated preclinical efficacy and increased immune response in mouse models (53). VERSATILE-002, a phase II trial, is studying the PDS0101 vaccine and pembrolizumab combination in recurrent/metastatic HPV-positive HNC.

##### Adoptive Cell Therapies

In addition to vaccines, several clinical trials have been initiated to study treatment strategies using adoptive T cell therapy against HPV-associated cancers. A phase I/II trial enrolled patients with HPV-positive tumors (N = 12) to undergo treatment with genetically engineered T cells with receptors targeting HPV-16 E6-expressing tumor cells as well as cyclophosphamide, fludarabine, and aldesleukin (NCT02280811) (54). Post-treatment results demonstrated anti-tumor response and decrease in tumor size, highlighting a role for adoptive T cell therapy in treating HPV-associated cancers. To that same effect, a phase I/II trial is currently recruiting patients to determine the dose and efficacy of engineered T cells targeting tumors cells with E7 protein (NCT02858310). Another phase II trial mimicked the study design of the previous trials with TILS treatment in combination with cyclophosphamide, fludarabine, and aldesleukin in HPV-associated cancers (NCT01585428). The trial showed an association between clinical response and HPV reactivity of the modified T cells as well as their presence in the peripheral blood (55). Potential biomarkers may predict response to novel therapeutics that utilize the patient’s cells will be important when utilizing this treatment method.

## Nasopharyngeal Carcinoma, EBV-Positive

NPC is a rare epithelial cancer type of the nasal cavity occurring most commonly in Southeastern Asia, China, Hong Kong, and Taiwan (8). It is characterized as an aggressive, locoregional disease that primarily affects males of Asian descent. Globally, there are approximately 129,079 new cases of NPC and 72,987 deaths from NPC annually, with a high incidence rate in males (3:1) (3, 56). Although early stage disease portends great 5-year survival rates of greater than 80%, stage IV metastatic disease is associated with poor survival rates of less than 25% (57, 58). NPC has been demonstrated to be highly sensitive to radiation therapy and chemotherapy, although resistance to therapy frequently occurs and patients with relapsed or metastatic disease inevitably recur with limited options for treatment (59–61).

### Treatment for Locally Advanced EBV+ NPC

EBV is an oncogenic, human-tropic γ-herpesvirus that infects >90% of the global population, mainly infecting epithelial and B-cells (62–64). After primary infection occurs, EBV establishes life-long residency in its host through establishment of latency in infected cells, although under different stimuli the virus can become reactivated and undergo lytic replication to result in the production of new virions (65). Similar to HPV, EBV miRNAs minimize antigen production in order to evade recognition by the immune system of the host, thus allowing for immune evasion and EBV latency in the host cells (**Figure 1**) (66). Over time, these EBV-affected cells can result in the development of various cancers. Both viral life stages have been associated with the development of several malignancies of lymphoid and epithelial cell origin including Burkitt’s lymphoma and Hodgkin lymphoma (65, 67). Of these malignancies, NPC holds the strongest association to EBV, with most NPC cases being EBV-positive (EBV+) (8, 62). The association between EBV and NPC was initially reported in 1973. Of the 129,000 cases of NPC globally diagnosed each year, ~97% are EBV+, with those occurring in high and intermediate incidence areas being 100% EBV+ and those in low incidence areas being 80% EBV+ (62, 68). Of the ~72,000 annual deaths attributed to NPC, ~97% are associated with EBV (68).

Treatment of locally advanced NPC typically involves multimodality therapy with concurrent chemoradiation treatment using platinum-based agents. Post-treatment plasma EBV levels have become a prognostic indicator of response and clinical outcomes in NPC (69, 70). To date, there are no licensed EBV-targeted strategies against EBV+ NPC.

Interest initially arose in utilizing individual patient’s immune cells to attack EBV-mediated cancers in order to leverage durable responses to this aggressive cancer. Ongoing clinical trials are exploring immunotherapy approaches using autologous and allogeneic EBV-specific T cells against NPC with promising interim results (71, 72).

### Treatment for Metastatic/Recurrent NPC

Unfortunately, recurrent or metastatic NPC is associated with poor outcomes and a median OS of 20 months (73). Standard-of-care treatment of recurrent/metastatic NPC involves platinum-based doublet chemotherapy. A milestone phase III clinical trial investigated the efficacy of platinum-based doublet chemotherapy treatment with cisplatin/gemcitabine or cisplatin/5-fluorouracil (5-FU) (74). 362 recurrent/metastatic NPC patients were randomized 1:1 to receive either combination. The ORR was 64% in the cisplatin/gemcitabine group versus 42% in the cisplatin/5-FU while median PFS was 7.0 months (cisplatin/gemcitabine) versus 5.6 months (cisplatin/5FU) (p <0.0001). This trial demonstrated statistically superior PFS with cisplatin/gemcitabine treatment, establishing the combination as standard first-line treatment for recurrent/metastatic NPC.

Although EBV-associated NPC results from EBV latency in the host cells due to immune evasion mechanisms by EBV, studies have been initiated to examine the potential of incorporating immunotherapy in NPC. Delord et al. presented the results from the NPC cohort in CheckMate 358, a phase I/II study evaluating nivolumab in virus-associated tumors (75). 24 patients with recurrent/metastatic NPC were enrolled, of which 88% were EBV positive tumors. The ORR was 20.8%, however the ORR was higher in patients that did not receive prior therapy in the metastatic setting (N = 5). With a median follow-up of 26 weeks, median PFS was 2.4 months while median OS was not reached. Another multicenter study (NCI-9742) examined the clinical efficacy of nivolumab in recurrent and metastatic NPC (76). Of the 44 patients that were enrolled in the trial, nine patients (20%) received nivolumab for over 12 months. The ORR was 20.5% (N = 9), the median PFS was 2.8 months, and the median OS was 17.1 months. The trial also examined the possible correlations of PD-L1 expression, human leukocyte antigens A and B expression, or EBV virus DNA levels with ORR and OS. They found that patients with greater than 1% PD-L1 expression were more likely to respond to nivolumab compared to PD-L1-negative NPC while EBV virus DNA levels had no impact. Interestingly, tumors that lacked human leukocyte antigens A and/or B expression were correlated with superior PFS versus tumors that expressed both antigens (30.9% versus 5.6%, p = 0.1). Overall, these trials demonstrated promising clinical results in treating this aggressive disease with nivolumab.

Keynote-028, phase Ib trial, evaluated the safety and efficacy of pembrolizumab 10 mg/kg every two weeks in PD-L1 positive (>1% expression) recurrent or metastatic NPC (77). With a median follow-up period of 20 months, the ORR was 25.9% of the 27 patients that were enrolled in the trial. By investigator review, the median PFS was 6.5 months and the median OS was 16.5 months. Although majority of patients were heavily pre-treated, pembrolizumab showed good safety profile and favorable anti-tumor activity in NPC patients with PD-L1 expression.

### Ongoing Trials and Development in NPC

#### Checkpoint Inhibitors in Locally Advanced NPC

Trials are underway to investigate the role of immune checkpoint inhibitors in the locally advanced setting given the encouraging results from metastatic/recurrent NPC trials. Lim et al. recently presented interim results from their phase II trial of nivolumab and ipilimumab in EBV+ locally advanced NPC (NCT03097939) (78). Eligible patients had EBV+ NPC, measurable blood EBV DNA levels, and a history of only one line of treatment. Of the 26 evaluable patients at the time of the presentation, the median duration of response was 5.9 months. The median PFS was 5.3 months with a median follow-up period of 10.6 months. Interestingly, they noted a difference in median PFS of EBV-low versus EBV-high patients (6.8 months versus 2.7 months, respectively). Overall, Lim at al. revealed encouraging preliminary results in combination PD-1 and CTLA-4 blockade in NPC patients. There are several ongoing trials evaluating monotherapy and combination immunotherapy strategies in locally advanced NPC, see **Table 2**.

**Table 2:** Ongoing clinical trials investigating monotherapy and combination immunotherapy strategies in locally advanced, recurrent, or metastatic NPC.

Study Title	NCT	Primary Endpoint	N	Phase	Experimental	Control	Definitive treatment
Sintilimab (PD-1 Antibody) and Chemoradiotherapy in Locoregionally-Advanced NPC	03700476	Failure-free survival	420	3 Randomized Multicenter	CRT with cisplatin, gemcitabine, and sintilimab	CRT with cisplatin and gemcitabine	CRT with cisplatin and gemcitabine
Neoadjuvant and Adjuvant Anti-PD-1 Antibody Toripalimab Combined with CCRT in NPC Patients	03925090	PFS	138	2 Randomized Single center	Neoadjuvant toripalimab followed by CRT with high dose cisplatin and toripalimab	Neoadjuvant placebo followed by CRT with high dose cisplatin	CRT with high dose cisplatin
Concurrent and Adjuvant PD-1 Treatment Combined with Chemo-radiotherapy for High-risk NPC	04453826	PFS	388	3 Randomized Multicenter	Induction cisplatin and gemcitabine followed by CRT with high dose cisplatin and camrelizumab	Induction cisplatin and gemcitabine followed by CRT with high dose cisplatin	Induction cisplatin and gemcitabine followed by CRT with high dose cisplatin
PACIFIC-NPC: Camrelizumab (PD-1 Antibody) After Chemoradiotherapy in Locoregionally Advanced NPC	03427827	Disease-free survival	400	3 Randomized Multicenter	Adjuvant camrelizumab	Observation	N/A
Programmed Death-1 (PD-1) Antibody Combined with IMRT in Recurrent NPC Patients	03907826	OS	212	3 Randomized Multicenter	Radiation therapy with concurrent and adjuvant toripalimab	Radiation therapy alone	Radiation therapy
PD-1 Knockout EBV-CTLs for Advanced Stage EBV Associated Malignancies (79)	03044743	Participants with adverse events	20	1/2 Single center Pilot study	Fludarabine and cyclophosphamide followed by PD-1 knockout EBV-CTLs and interleukin-2	N/A	Fludarabine and cyclophosphamide
EBV-TCR-T YT-E001)for Patients With EBV-positive Recurrent or Metastatic NPC	03648697	Participants with adverse events	20	1/2 Single center Pilot study	Fludarabine and cyclophosphamide followed by EBV-TCR-T (YT-E001) cell infusion	N/A	Fludarabine and cyclophosphamide
LMP2 Antigen-specific TCR T-cell Therapy for Recurrent and Metastatic NPC Patients	03925896	Maximum tolerated dose	27	1 Single center	LMP2 Antigen-specific TCR T cells	N/A	N/A

N/A, not applicable.

#### Checkpoint Inhibitors in Recurrent/Metastatic NPC

In the recurrent/metastatic setting, camrelizumab is also under investigation in combination with cisplatin and gemcitabine in a phase III clinical trial (NCT03707509). Prior results were published from two phase I trials where previously treated recurrent or metastatic NPC patients received camrelizumab monotherapy while treatment naïve patients received six cycles of camrelizumab, cisplatin, and gemcitabine with adjuvant maintenance camrelizumab (80). In the camrelizumab monotherapy trial versus the camrelizumab combination trial, the ORR was 34% versus 91%, demonstrated promising clinical activity. Interim results from a phase II trial of patients randomized to receive spartalizumab (PDR001), a humanized anti-PD-1 IgG4 mAb, or chemotherapy (NCT02605967) were recently presented (81). The trial did not reach its primary endpoint of PFS (median PFS of 1.9 months in the spartalizumab arm versus 6.6 months in the chemotherapy arm). However, the duration of response at 12 months in patients responding in the spartalizumab group was 61.0%. While spartalizumab monotherapy did not improve PFS compared to chemotherapy regimens, a subset of patients could potentially benefit with durable response to the monotherapy treatment.

Keynote-122, an active phase II study of pembrolizumab versus chemotherapy (capecitabine, gemcitabine, or docetaxel) in platinum-pretreated recurrent or metastatic NPC (NCT02611960) (82), was initiated due to results from the phase IB Keynote-028 reporting an ORR of 25.9% as well as promising median PFS/OS in heavily pre-treated NPC patients treated with pembrolizumab (77). A phase III trial of tislelizumab, a humanized monoclonal antibody against PD-1, with cisplatin and gemcitabine versus chemotherapy alone is currently underway (NCT03924986) after preliminary results from the phase II trial showed 80% of patients (N = 9 out of 15) achieving a partial response or stable disease with monotherapy tislelizumab treatment (83). Results from a trial examining an EBV-specific immunotherapy drug (NCT00834093) demonstrated a poor ORR, a median PFS of 2.2 months, and a median OS of 16.7 months (84). Please refer to **Table 2** for a list of ongoing trials recruiting metastatic/recurrent NPC patients to investigate the role of novel monotherapy and combination immunotherapy drugs.

#### Vaccine Therapy in NPC

EBV+ NPC cancers express a select set of latent EBV antigens, which offers an excellent opportunity for targeted therapeutics (85–87). NPC is characterized by the expression of the latent antigen EBNA1, responsible for maintenance of the viral genome in infected cells, and the latent antigen LMP2, which supports proliferation, survival and migration of infected cells (67, 88–90). Currently, these two antigens are being tested together as components of a therapeutic vaccine candidate in phase I/II clinical trials against EBV+ NPC, and have been shown to be immunogenic and able to elicit EBV-specific CD4+ and CD8+ T-cell responses (91, 92).

#### Adoptive Cellular Therapy in NPC

The ability to target EBV as a therapeutic option represents an important milestone, especially in light of limited treatment strategies available for recurrent/metastatic EBV+ NPC. Adoptive cellular therapy utilizes individual patient’s immune cells to attacked EBV-mediated cancers including NPC. VANCE, a phase III trial is currently underway investigating carboplatin and gemcitabine doublet chemotherapy with infusions of autologous EBV-specific cytotoxic T cells in advanced NPC (NCT02578641). This trial was initiated based on the phase II trial results which demonstrated superior survival outcomes with 2-year OS at 62.9%, a median PFS of 7.6 months, and a median OS of 29.9 months in 35 recurrent/metastatic NPC patients who received the combination therapy (93). They also found that high EBV-DNA levels were correlated with high tumor burden and poor prognosis. An ongoing phase Ib/2 trial (NCT03769467) is assessing the tolerability and anti-tumor activity of combination tabelecleucel, an allogeneic T-cell immunotherapy, and pembrolizumab in platinum-treated recurrent/metastatic EBV+ NPC (94). The primary objective of the phase Ib portion is to identify the maximum tolerated dose and any dose-limiting toxicities while the phase II portion’s aims are safety and ORR. Additional trials are currently recruiting patients to investigate other adoptive T cell therapies in recurrent/metastatic NPC (**Table 2**).

## Conclusions

There has been vast work in employing the immune system in HNSCC. Most immune therapy clinical trials include a very heterogenous population of HNSCC patients and may only stratify OPSCC and NPC based on viral etiology resulting in small subgroups hence, direct comparisons of viral and non-viral-related HNSCC are difficult to compare statistically. Given the implications of immune dysfunction in viral-associated malignancies, treatment targeting the immune system is a reasonable option for clinical success but may be challenging. Most clinical work with immune therapy has been done in the metastatic setting with some encouraging results. There are subsets of patients who respond very well while others have no response at all. Lack of response is likely multifactorial and may be due to the difficulty in leveraging the immune system in viral-associated HNCs that employ various mechanisms of immune evasion. There are many levels of investigation which need attention to ensure success with these strategies including the identification of: who is most likely to respond, the confounding factors of non-responsiveness, which immune and non-immune targets should be addressed, and which strategies alone or together have a higher likelihood of eradicating the cancer. Further work is needed to identify the line of therapy which is most conducive for immune therapies. Many of these immune targeted strategies are currently under evaluation in earlier settings and including, curative intent treatments of virally induced OPSCC and NPC. We believe immune resistance is multifactorial and the biological properties of this disease need to be further delineated to better understand mechanisms of immune resistance. Future directions involve combination regimens including doublet immune checkpoint inhibitors, immune checkpoint inhibitors and vaccines, as well as adoptive cellular therapy.

## Author Contributions

RP, YX, MA, and VV all contributed to the conceptualizing, writing, and editing of this work. All authors contributed to the article and approved the submitted version.

## Conflict of Interest

VV reports consultant/advisory fees for AstraZeneca, Bristol-Myers Squibb, and Genentech. MA reports consultant/advisory fees for Lilly, Adaptimmune, Regeneron, and AstraZeneca, and speaker bureau participation for Bristol-Myers Squibb and Bayer.

The remaining authors declare that the research was conducted in the absence of any commercial or financial relationships that could be construed as a potential conflict of interest.
